# Short environmental enrichment in adulthood reverses anxiety and basolateral amygdala hypertrophy induced by maternal separation

**DOI:** 10.1038/tp.2015.217

**Published:** 2016-02-02

**Authors:** A S Koe, A Ashokan, R Mitra

**Affiliations:** 1 School of Biological Sciences, Nanyang Technological University, Singapore, Singapore

## Abstract

Maternal separation during early childhood results in greater sensitivity to stressors later in adult life. This is reflected as greater propensity to develop stress-related disorders in humans and animal models, including anxiety and depression. Environmental enrichment (EE) reverses some of the damaging effects of maternal separation in rodent models when provided during peripubescent life, temporally proximal to the separation. It is presently unknown if EE provided outside this critical window can still rescue separation-induced anxiety and neural plasticity. In this report we use a rat model to demonstrate that a single short episode of EE in adulthood reduced anxiety-like behaviour in maternally separated rats. We further show that maternal separation resulted in hypertrophy of dendrites and increase in spine density of basolateral amygdala neurons in adulthood, long after initial stress treatment. This is congruent with prior observations showing centrality of basolateral amygdala hypertrophy in anxiety induced by stress during adulthood. In line with the ability of the adult enrichment to rescue stress-induced anxiety, we show that enrichment renormalized stress-induced structural expansion of the amygdala neurons. These observations argue that behavioural plasticity induced by early adversity can be rescued by environmental interventions much later in life, likely mediated by ameliorating effects of enrichment on basolateral amygdala plasticity.

## Introduction

Adversity in early postnatal life has a lasting negative impact on behavioural and emotional functioning of individuals.^1, 2, 3^ Compelling evidence suggests that rodent maternal separation (MS), now widely used to mimic early-life stress in human infants, leads to hyper-reactivity of the hypothalamic−pituitary−adrenal axis in adulthood.^4, 5, 6^ This is accompanied by anxious or depressive-like behaviour or cognitive deficits in some instances.^7, 8, 9, 10^ In addition, these effects have been suggested to be downstream of neuroplasticity alterations. In particular, early-life stress leads to reductions in hippocampal neurogenesis^11, 12, 13^ as well as retraction of dendrites and spine density of the hippocampus^14, 15, 16, 17, 18^ and medial prefrontal cortex (mPFC).^17, 19, 20^ A single episode of MS for 24 h at postnatal day 3 does not affect dendritic complexity in the basolateral amygdala (BLA).^21^ It is not known if repeated MS results in BLA structural plasticity in parallel to its observed potentiation of anxiety. This is an important gap in knowledge because dendritic and spine changes in the BLA are central to stress-induced anxiogenesis.^22, 23, 24^

While the effects of adversity in early life possess a degree of permanence, peripubertal environmental enrichment (EE) has been shown to reverse its effects on anxiety,^25, 26^ depression^27, 28^ and learning deficits.^27, 28, 29^ While reversibility of developmental adversity has been demonstrated in young animals, it is presently undetermined if EE in adulthood is able to reverse the persistent effects of early-life stress long after the event. This is an important question because, if effective, an adult intervention can provide the opportunity to loosen the health burden of the temporally distant adverse past.

In this background, we experimentally test whether MS results in BLA plasticity during adulthood, and if adult EE rescues the effects of MS on anxiety-like behaviour and BLA plasticity.

## Materials and methods

### Experimental animals

Wistar rats were procured from InVivos, Singapore. Animals were maintained and mated in Nanyang Technological University vivarium on a 12:12-h light−dark schedule (lights on at 0700 h). Sires were removed from the cage once pregnancy was confirmed. All litters were weaned on postnatal day 21 (PN21). Male pups were group-housed after weaning until 6 weeks of age, when they were assigned to a density of two animals per cage. All procedures were approved by the Nanyang Technological University Institutional Animal Care and Use Committee.

### Maternal separation

On PN2, litters were randomly assigned to undergo MS, or to be reared under animal facility rearing (AFR) conditions. MS was carried out from PN2 to PN14, inclusive, and consisted of daily separation of whole litters from their dams for 3 h (0900–1200 h). First, dams were removed from the home cage and placed in a clean cage in the same room. Pups were then removed from the nest one at a time and placed together in a smaller cage and kept in a quiet, separate room on a heating pad to maintain body temperature. At the end of the separation period, pups were returned to the home cage, followed by the dam. On PN2, PN9 and PN14, the pups and dam were placed in a clean home cage. AFR litters were left undisturbed except for cage change on PN2, PN9 and PN14. From PN15 to PN21, litters remained housed with dams until weaning. A total of nine litters were used (four MS, five AFR), generating a total of 49 male pups. Body weight was measured once a week from weaning (PN21) to the end of the experiment.

### Environmental enrichment

On PN56, male offspring from AFR (*n*=23) and MS (*n*=26) litters were pseudorandomly divided into two housing subgroups: EE (AFR-EE, *n*=11; MS-EE, *n*=14) or standard housing (AFR-Standard, *n*=12; MS-Standard, *n*=12). Sample size was estimated based on historical variance in the laboratory for similar experiments. Housing conditions were maintained for the rest of the experiment. EE animals were housed in groups of 2–4 in a complex highly sensory environment consisting of a large, three-level cage (72 × 51 × 110 cm^3^) containing a variety of cylindrical plastic pipes, nesting material, toys, hanging platforms, baskets and treats. The objects within the EE cage were rearranged and renewed every four days. Non-enriched animals were housed in pairs in individually ventilated standard laboratory cages (37 x 22 x 18 cm^3^).

### Behavioural testing

Following 14 days of EE/standard housing, animals were subjected to the home cage emergence test at PN70, and subsequently to the elevated plus maze (PN74) to measure anxiety. All behavioural procedures commenced at approximately 1000 h, with a 30-min habituation to ambient lighting conditions (number of animals tested for behavioural experiments mentioned in the previous section). The same cohort was used for all behavioural tests in the sequence specified below. Animals stayed in the EE for 14 days preceding testing and remained so until they were killed (Supplementary Figure 1). The duration of EE before behavioural testing was guided by the ability of a similar duration of EE to rescue the behavioural effects of stress when both EE and stress were provided in adulthood.^30^

### Home cage emergence test

In the home cage emergence test, two standard laboratory rat cages with lids removed were placed 10 cm apart, with a grid placed against one of the edges of one (home cage) leading out into the other. Rats were placed in the home cage, and latency to leave the cage was measured. Leaving the cage was operationally defined when all four paws were on the grid. If the rat did not emerge from its home cage within 10 min, the session was ended and the rat was given a maximum score of 600 s. The test was carried out in dim light conditions (3−4 lux in both cages).

### Elevated plus maze

The elevated plus maze apparatus consisted of a raised plus-shape maze (60 cm from ground) with two opposite arms enclosed with walls (75 × 11 × 26 cm^3^) and the other two arms exposed (75 × 11 cm^2^). Dim light was shone directly onto each open arm (6 lux). Each rat was placed in the centre square of the maze and allowed to explore freely for 5 min. All trials were video-recorded and manually analysed to quantify the percentage open-arm time and open-arm entries, relative to the sum of open and enclosed arm exploration. As a measure of risk assessment, the number of head dips was also quantified. The experimenter analysing the record was blind to animal number and treatment.

### Open-field test

Animals were placed in an open circular arena (radius=120 cm, trial duration=300 s, diffused dim lighting). Time spent in the centre of the field was quantified as the reciprocal proxy of the anxiety (centre defined as a concentric circle to the arena with 0.33 m radius). Total distance travelled during the trial was also quantified as a measure of locomotion.

### Brain collection

On PN84, rats were killed by decapitation. Terminal trunk blood was collected, serum was separated and used for estimation of corticosterone concentration using a commercial EIA kit (Enzo Life Sciences, ADI-900-097, Farmingdale, NY, USA). The brain was quickly removed and processed for Golgi staining using the FD Rapid GolgiStainTM kit (FD Neurotechnologies, Columbia, SC, USA). Coronal sections (100 μm thickness) were then cut using a cryostat (Leica CM3050-S, Leica Biosystems, Wetzlar, Germany) and collected on gelatinized slides. Sections were counterstained in 0.25% cresyl violet solution and coverslipped using Permount mounting medium (Fisher Scientific, Singapore).

### Dendritic morphology of BLA neurons

A random proportion of animals from each group was used for morphological analysis (AFR-Standard: *n*=6; AFR-EE: *n*=9; MS-Standard: *n*=9; MS-EE: *n*=9). Complete stellate or pyramidal-like neurons in the BLA, consisting of the lateral and basal nuclei, were selected for tracing using a microscope (Olympus BX43, Tokyo, Japan, × 40 objective lens) with the aid of a camera lucida. For each animal, 10−11 neurons were drawn to yield a representative sample of BLA neurons for each group. Custom-designed macros embedded in ImageJ (http://rsb.info.nih.gov/ij/) were used for analysis of scanned images to quantify total dendritic length and total number of branch points. All neurons were drawn and analysed by an experimenter blind to treatment. The codes were not broken until quantification for dendritic length and spine density was concluded. Dendritic length and branch points of prelimbic mPFC neurons were quantified in the same cohort of animals.

### Spine density of BLA neurons

Using the same microscope (Olympus BX43, 1.3 numerical aperture, × 100 objective lens), all protrusions from dendrite, irrespective of morphological characteristics, were counted as spines. Dendrites directly originating from the cell soma were classified as primary dendrites, and the first branch emerging from the primary dendrite was classified as the secondary dendrite. Starting from the origin of the branch, and continuing away from the cell soma, spines were counted along a 60-μm stretch of the dendrite. Spine density was quantified in 6–10 neurons per animal to yield a representative sample of BLA neurons for each group.

### Statistical analyses

Normality for behavioural and morphological end points was examined using the Shapiro−Wilk test. Several end points exhibited significant departure from normality (Table 1). Consequently non-parametric statistics was used for intergroup comparisons (Kruskal–Wallis one-way analysis of variance). When significant intergroup differences were indicated, group-wise *post-hoc* comparisons were conducted using Mann−Whitney *U* test (two-tailed). The AFR group was compared with the MS group, either in the presence or in the absence of EE. Resultant type 1 error probabilities were adjusted for multiple comparisons using Bonferroni correction. Figures depict median and interquartile range. Effect size was calculated using Cliff's delta, a non-parametric statistic.^31^ All analyses were performed using IBM SPSS statistics 21 (Armonk, NY, USA) or GraphPad Prism 6 software (La Jolla, CA, USA).

## Results

There were no significant differences in litters randomly chosen to undergo MS and AFR, in terms of both litter size (Student's *t*-test; *t*_(7)_ =1.49, *P*=0.18) and number of male pups (*t*_(7)_ =1.76, *P*=0.12). At weaning (PN21), male pups exposed to MS weighed significantly less compared to the AFR group (AFR: 45.50±0.7 g; MS: 42.60±1.1 g; *t*_(47)_=2.20, *P*=0.03). MS-treated animals continued to weigh less than non-MS animals into the adulthood (Supplementary Figure 2). Several behavioural and structural end points exhibited non-normal frequency distribution (Table 1). A rank transformation of data failed to institute normality. In view of non-normal distribution, all subsequent data analysis employed non-parametric alternatives.

### Home cage emergence test

**Figure 1:**
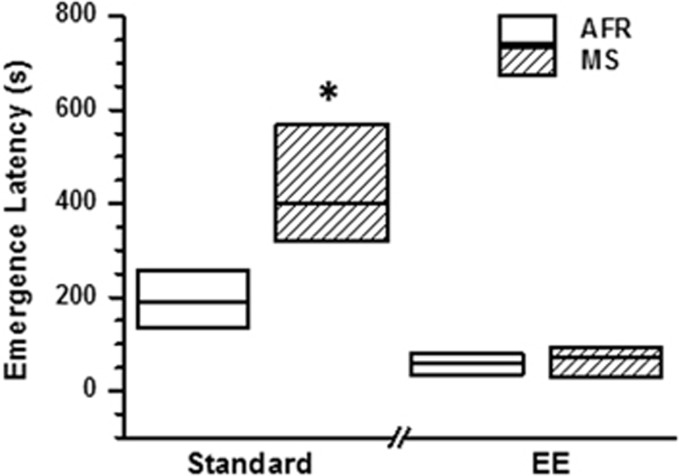
Effect of maternal separation (MS) and environmental enrichment (EE) on latency to emerge from the home cage. The figure depicts the median and interquartile range (between 25th to 75th percentiles) of emergence latency. *P<0.01 for comparison between MS and animal facility rearing (AFR) groups; Mann−Whitney *U* test, Bonferroni correction applied for multiple (*X*^2^) comparisons. *N*=12 animals for AFR-Standard, 12 for MS-Standard, 11 for AFR-EE and 14 for MS-EE.

Latency to emerge from home cage was quantified as a reciprocal end point for anxiety-like behaviour (Figure 1). Kruskal−Wallis test revealed significant intergroup differences (*χ*^2^=25.3, df=3, *P*=0.0001). Subsequent *post-hoc* analysis revealed significantly higher emergence latency in MS rats compared to AFR when both groups were housed in standard conditions during adulthood (Figure 1; Mann−Whitney *U* test: |*z*|=2.40, *P*=0.032 *after* Bonferroni correction for multiple comparisons; *n*=12 for AFR-Standard and MS-Standard). The 25th percentile of MS-Standard animals was observed to be greater than the 75th percentile of the AFR-Standard group, suggesting a robust increase in emergence latency due to MS (effect size in Table 1). In contrast, MS did not significantly increase emergence latency in the presence of EE (Figure 1 and Table 1; |*z*|=0.27, *P*=0.784 *before* Bonferroni correction; *n*=11 for AFR-EE and 14 for MS-EE).

### Elevated plus maze

**Figure 2:**
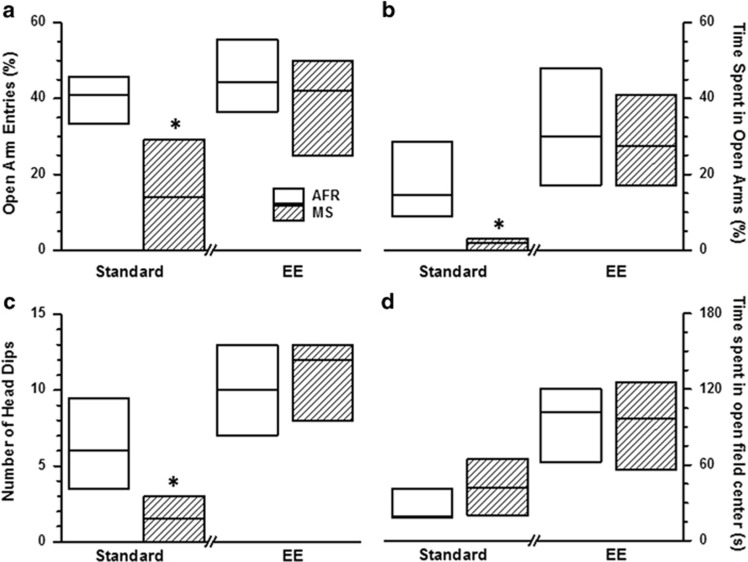
Effect of maternal separation (MS) and environmental enrichment (EE) on percentage open-arm entries (**a**), percentage open time (**b**), number of head dips (**c**) in elevated plus maze and time spent in the centre of an open-field arena (**d**). Percentage open-arm exploration is quantified relative to the sum of open- and enclosed-arm exploration, in %. *P<0.01 for comparison between MS and animal facility rearing (AFR) groups; Mann−Whitney *U* test with Bonferroni correction. *N* is similar to Figure 1. *N*=14 animals for AFR-Standard, 10 for MS-Standard, 15 for AFR-EE and 10 for MS-EE for D.

The elevated plus maze was used as a second assessment for anxiety-like behaviour, with decreased open-arm entries and time indicative of heightened anxiety. Kruskal−Wallis test revealed significant intergroup differences for percentage open-arm entries (relative to sum of open and enclosed arms, % *X*^2^=15.7, df=3, *P*=0.0013) and percentage open-arm time (*X*^2^=24.7, df=3, *P*=0.0002). Congruent to emergence latency from home cage, MS caused robust decrease in percentage open-arm entries when housed in standard conditions during adulthood (Figure 2a; |*z*|=3.00, *P*=0.006 *after* Bonferroni correction). In contrast, differences between MS and AFR groups were not statistically significant when animals were housed in enriched environment (|*z*|=1.07, *P*=0.57 *after* Bonferroni correction). Similarly, MS significantly decreased percentage open-arm time in the absence of EE (Figure 2b; |*z*|=3.65, *P*=0.001 *after* Bonferroni correction), but not in the presence of EE (|*z*|=0.06, *P*=0.956 *before* Bonferroni correction).

In addition to quantifying entries in open and enclosed arms, we also measured the number of head dips as a measure of risk assessment (Figure 2c). Kruskal−Wallis test revealed significant intergroup differences for this end point (*X*^2^=28.6, df=3, *P*<0.0001). MS treatment resulted in a significant decrease in risk assessment under standard housing conditions (Figure 2c; |*z*|=3.61, *P*=0.001 *after* Bonferroni correction). The effect of MS on head dips was absent when animals were housed under EE (|*z*|=0.80, *P*=0.850 *after* Bonferroni correction).

Estimates of effect size (Table 1) demonstrated a robust effect of MS on all parameters during elevated plus maze paradigms. Thus, the effect size for anxiety parameters exceeded a relatively robust level of 0.65 after exposure to MS. This is also borne out by the observations that, in all parameters, the 75th percentile of MS animals was lower than the 25th percentile of the AFR group (Figure 2). Adult provision to EE substantially diminished the effect sizes for MS exposure (Cliff's delta between −0.25 to 0.25; Table 1).

### Open-field test

Time spent in the centre of the open-field arena and total locomotion in the arena were quantified. Kruskal−Wallis test revealed significant intergroup differences for occupancy in the centre (Figure 2d; *X*^2^=22.8, df=3, *P*<0.0001). Subsequent *post-hoc* analysis did not reveal statistically significant effect of MS on time spent in the centre, in either absence (*P*>0.2) or presence (*P*>0.7) of EE. In the non-MS group of animals, EE significantly increased time spent in the centre of the arena (*P*<0.001 *after* Bonferroni correction). Distance travelled in the open field did not show significant intergroup differences (*X*^2^=0.98, df=3, *P*>0.8), suggesting comparable locomotion between experimental groups.

### Dendritic morphology of BLA neurons

Two parameters, total dendritic length and number of branch points, were quantified in 334 BLA neurons (8−11 neurons per animal, average for each individual animal used for statistical analysis). Kruskal−Wallis test revealed significant intergroup differences for both total dendritic length (μm; *X*^2^=8.5, df=3, *P*=0.037) and total number of branch points (*X*^2^=19.1, df=3, *P*<0.003).

**Figure 3:**
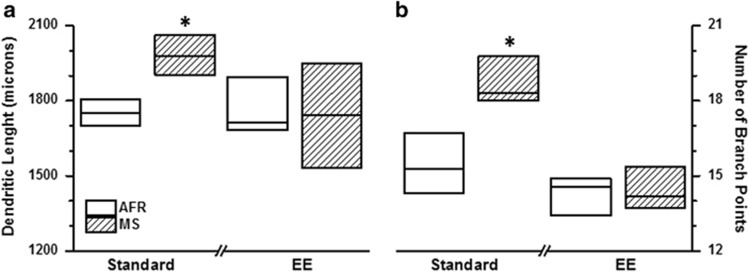
Effect of maternal separation (MS) and environmental enrichment (EE) on total dendritic length (**a**, μm) and number of branch points (**b**) of principal basolateral amygdala neurons. *P<0.01 for comparison between MS and animal facility rearing (AFR) groups; Mann−Whitney *U* test with Bonferroni correction. *N*=6 animals for AFR-Standard, 9 animals for all other groups; the value for each individual animal was derived as the average of 8–11 unique neurons.

MS resulted in a significant hypertrophy of BLA neurons. This was evident as increase in both total dendritic length (Figure 3a; |*z*|=2.47, *P*=0.026 *after* Bonferroni correction) and number of branch points (Figure 3b; |*z*|=2.95, *P*=0.006 *after* Bonferroni correction). MS-induced hypertrophy did not reach statistical significance when animals were provided with EE (|*z*|=0.22, *P*=0.863 *before* Bonferroni correction for both dendritic length and branch points). In the absence of EE, the 25th percentile of MS animals was greater than the 75th percentile of the AFR group (Figures 3a and b), resulting in a robust effect size of >0.75 (Table 1). This increase in dendritic parameters was normalized in the presence of EE (effect size=0.06).

**Figure 4:**
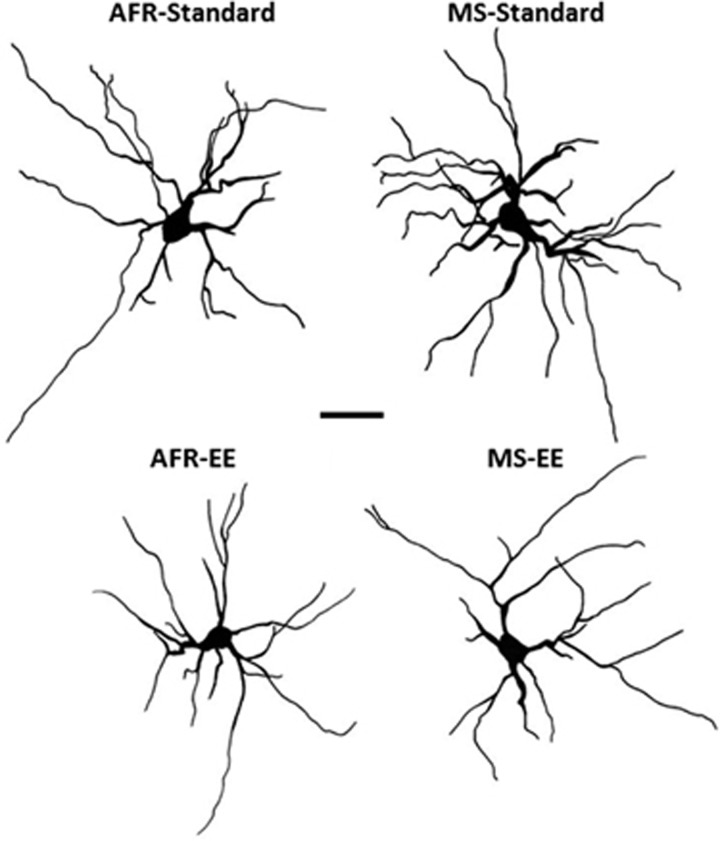
Representative camera lucida drawings of Golgi-impregnated basolateral amygdala neurons from animals exposed to maternal separation and/or environmental enrichment. Scale bar=50 μm.

Figure 4 depicts camera lucida traces of representative neurons.

Dendritic length and branch points of prelimbic mPFC neurons were quantified in the same cohort of animals (8 neurons per animal, average for each individual animal used for statistical analysis; Table 1). Kruskal−Wallis test did not reveal significant intergroup differences for both total dendritic length (Supplementary Figure 3; μm; *X*^2^=1.74, df=3, *P*=0.627) and total number of branch points (*X*^2^=1.39, df=3, *P*=0.708).

### Spine density of BLA neurons

**Figure 5:**
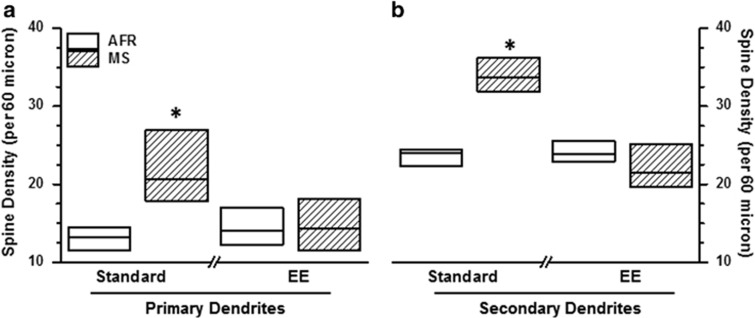
Effect of maternal separation (MS) and environmental enrichment (EE) on the spine density in primary (**a**, per 60 μm) and secondary (**b**) dendrites of principal BLA neurons. *P<0.01 for comparison between MS and animal facility rearing (AFR) groups; Mann−Whitney *U* test with Bonferroni correction. *N*=6 animals for AFR-Standard, 8 animals for all other groups; the value for each individual animal was derived as the average of 8–11 unique neurons.

The number of spines was quantified in a 60-μm segment of primary and secondary dendrites (Figure 5). Kruskal−Wallis test revealed significant intergroup differences for both primary (number of spines per 60 μm; *X*^2^=7.7, df=3, *P*=0.05) and secondary dendrites (*X*^2^=13.5, df=3, *P*<0.004). In cases of both primary and secondary dendrites, *post-hoc* analysis indicated significant increase in spine density due to MS exposure (Figures 5a and b; |*z*|>2.32, *P*<0.04 *after* Bonferroni correction). The MS-induced increase in spine density was renormalized when animals were exposed to EE during adulthood (|*z*|<0.85, *P*>0.40 *before* Bonferroni correction). Consistent with dendritic parameters, the 25th percentile of MS-Standard animals exceeded the 75th percentile of the AFR-Standard group, suggesting a robust experimental effect (Table 1; effect size>0.75). In contrast, exposure to EE reduced the effect sizes for MS treatment.

### Serum corticosterone concentration

Serum concentration of stress hormone corticosterone was quantified in trunk blood obtained during death. Corticosterone concentration did not show significant intergroup differences (*X*^2^=0.80, df=3, *P*>0.8). This suggests that stress hormones circulating at the baseline in adulthood were not different between experimental groups (Supplementary Figure 4).

## Discussion

The data presented in this report demonstrate that MS in early life leads to structural changes in the BLA during adulthood. BLA is a critical brain region in the generation and maintenance of conditioned fear and generalized anxiety (succinctly reviewed in Fanselow and LeDoux,^32^ Pare,^33^ Nathan *et al.*^34^ and Boyle^35^). For example, traumatic stress in the form of predator exposure in adult rats causes long-lasting anxiety. This anxiogenesis is dependent on the BLA^36^ and secretion of stress hormones.^37, 38^ Similarly, lesions of the BLA rescue the effects of stress hormones on a variety of memory processes.^39, 40, 41, 42^

Prior work has shown that chronic stress or exogenous glucocorticoids during adulthood precipitate dendritic hypertrophy of the BLA projection neurons.^23, 24^ Stress also enhances spine density ^22^ and reduces synaptic inhibition on these neurons.^43^ These observations suggest that stress or stress hormones act within the BLA, causing structural expansion and increased excitability. Such stress-induced facilitation consequently leads to greater anxiety, given the pivotal role of the BLA in the generalized fear. This suggestion is buttressed by the observation that augmentation of BLA dendrites co-occurs with more anxiety;^22, 23, 44^ blockade of stress hormonal action in or experimental reduction of dendritic length within the BLA reduces anxiety;^45, 46^ and inter-individual variation in stress-induced anxiety co-elutes with the dendritic architecture of BLA neurons.^47^ In light of these observations, we suggest that structural changes in BLA projection neurons long after MS results in the increased anxiety reported here and elsewhere.

Previous studies have reported effects of MS on the dendritic architecture of PFC projection neurons.^17, 20^ Adolescent and postpubertal rats exhibit reduced dendritic complexity and lower spine density in these neurons after MS. Similarly, MS suppresses dendritic complexity of neurons in the hippocampus and nucleus accumbens. These observations are in marked contrast to dendritic expansion and denser spines reported in the present study. Interestingly, chronic stress during adulthood also results in contrasting changes in the BLA vis-à-vis the hippocampus or the PFC.^22, 23, 24, 48, 49, 50^ Congruently, MS causes contrasting changes in the behaviours mediated by these structures. For example, MS results in deficits of hippocampus dependent spatial memory and PFC dependent extinction recall of conditioned fear.^16, 51^ In contrast, MS increases anxiety-like behaviour consistent with the role of the BLA in anxiety. We posit that the differential effects of MS on BLA dendrites, compared to hippocampal and prefrontal neurons, underlie the disparate effects of early-life stress on anxiety and memory processes. Furthermore, a single acute session of MS does not result in the dendritic changes in BLA.^21^ Thus, the chronic and repetitive aspects of MS employed here are likely necessary for the BLA's structural plasticity.

EE is reported to have positive effects on a variety of emotional and cognitive parameters (reviewed in Alwis and Rajan,^52^ Arai and Feig,^53^ Eckert and Abraham,^54^ Hannan,^55^ Pang and Hannan,^56^ and van Praag *et al.*^57^). In case of MS effects, peripubertal EE immediately after weaning rescues the effects of MS on endocrine reactivity to acute stress.^25^ Thus, MS increases stress-induced release of corticosterone from adrenal glands and enhances the amount of corticotrophin-releasing factor present in the hypothalamus, suggesting greater sensitivity to acute stress during adulthood. Fifty-days-long peripubertal EE starting at PN21 renormalizes such stress reactivity, in parallel to also rescuing MS-induced anxiety in an open-field test. Similarly, peripubertal EE also rescues the cognitive effects of low maternal care in rats.^29^ In contrast to these reports, the present observations suggest that a relatively short EE spanning 2 weeks and starting much later in adult life is sufficient to rescue both behavioural consequences of MS and underlying neuronal changes. Thus, the presence of a long environmental intervention during adolescence is sufficient, but not necessary, for the rescue. Similar renormalization can also be achieved outside the critical peripubertal window and through a shorter intervention.

The current data suggest that circuits underlying emotional behaviour maintain a degree of plasticity in adulthood, such that positive and stimulating environments can overcome the effects of adversity applied during a highly sensitive period of brain development. In addition, our findings propose neuroplasticity in the amygdala as a likely candidate in mediating the reversal of MS effects. Altered neuronal morphology could therefore be a phenotype for increased risk of affective behaviour following early-life stress, and may provide a potential target for treatment of mental disorders associated with early adversity. At the same time, our findings also propose environmental stimulation in adulthood as a possible therapy for neuropsychiatric conditions associated with early-life stress, particularly those known to involve altered amygdala structure and function.

## Figures and Tables

**Table 1:** Tests for normality and effect size

*End point*	*Shapiro−Wilk test*			*Cliff's delta statistics* a	
	*Statistics*	*df*	P	*No EE*	*With EE*
*Home cage emergence*					
Emergence latency	0.820	49	>0.001	−0.597	0.052
					
*Elevated plus-maze*					
Percentage open entries	0.923	49	0.003	+0.653	+0.234
Percentage open time	0.940	49	0.015	+0.833	+0.000
Number of head dips	0.955	49	0.057	+0.764	−0.247
					
*BLA dendrites*					
Total dendritic length	0.975	33	0.625	−0.778	+0.062
Total branch points	0.921	33	0.019	−0.926	+0.062
					
*BLA spine density*					
Primary dendrites	0.894	30	0.006	−0.750	+0.031
Secondary dendrites	0.911	30	0.016	−0.833	+0.375
					
*Prefrontal cortex dendrites*					
Total dendritic length	0.987	30	0.965	+0.400	−0.238
Total branch points	0.930	30	0.048	−0.200	−0.079

Abbreviations: BLA, basolateral amygdala; EE, environmental enrichment.

a Non-parametric estimate of effect size. Animal facility rearing (AFR) versus maternal separation (MS). Ranges from −1 (AFR<MS) to +1 (AFR>MS).
