# Supplementary figures and images for: Short environmental enrichment in adulthood reverses anxiety and basolateral amygdala hypertrophy induced by maternal separation
Source: Transl Psychiatry. 2016 Feb 2;6(2):e729–. doi: 10.1038/tp.2015.217 (PMC4872421; doi:10.1038/tp.2015.217)

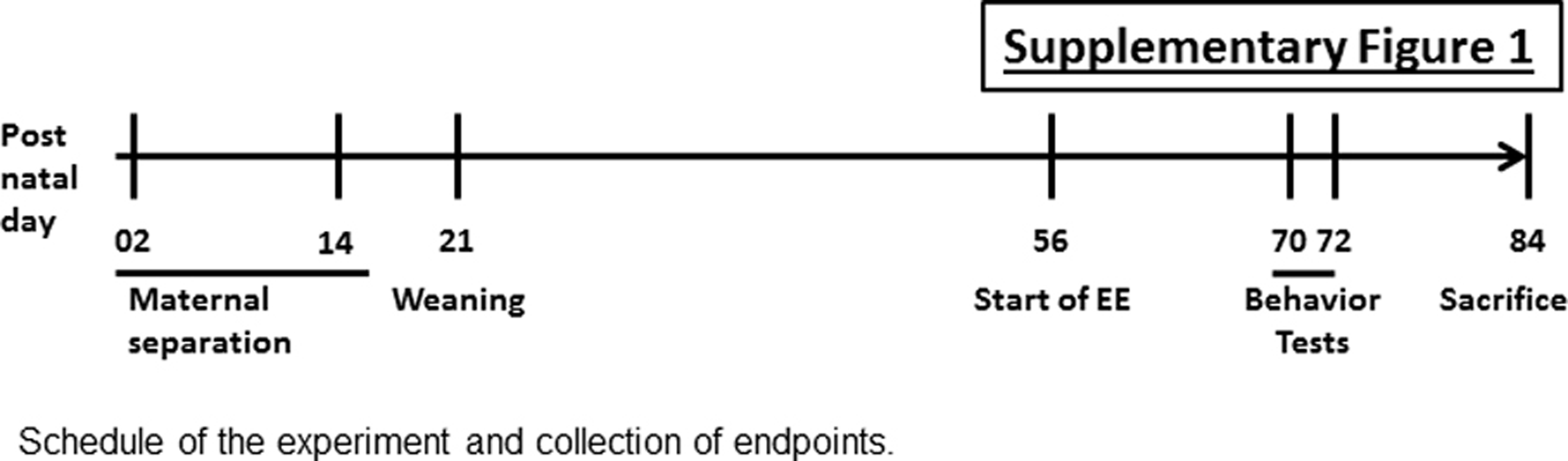

Supplement: Supplementary Figure 1 [file tp2015217x1.tif]

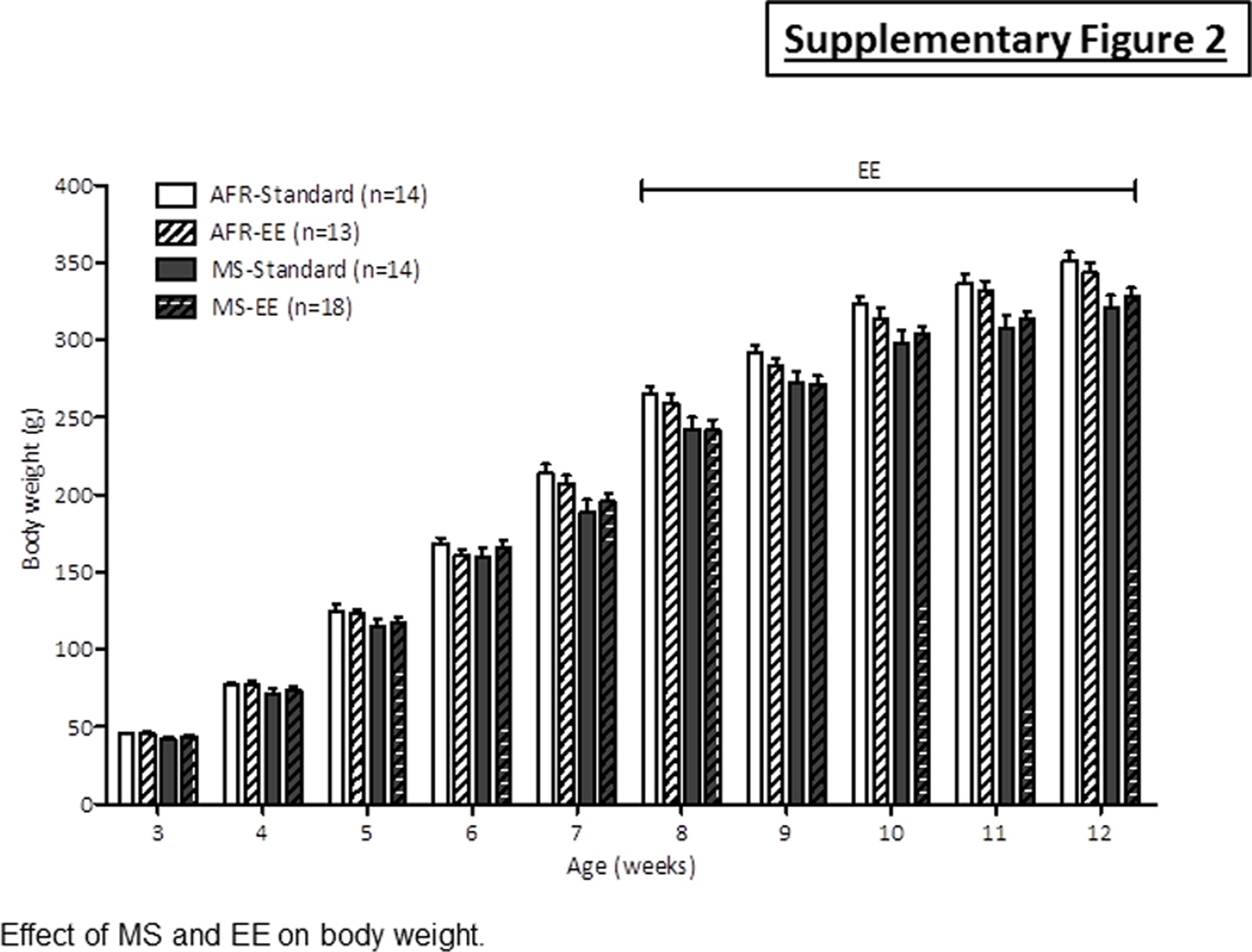

Supplement: Supplementary Figure 2 [file tp2015217x2.tif]

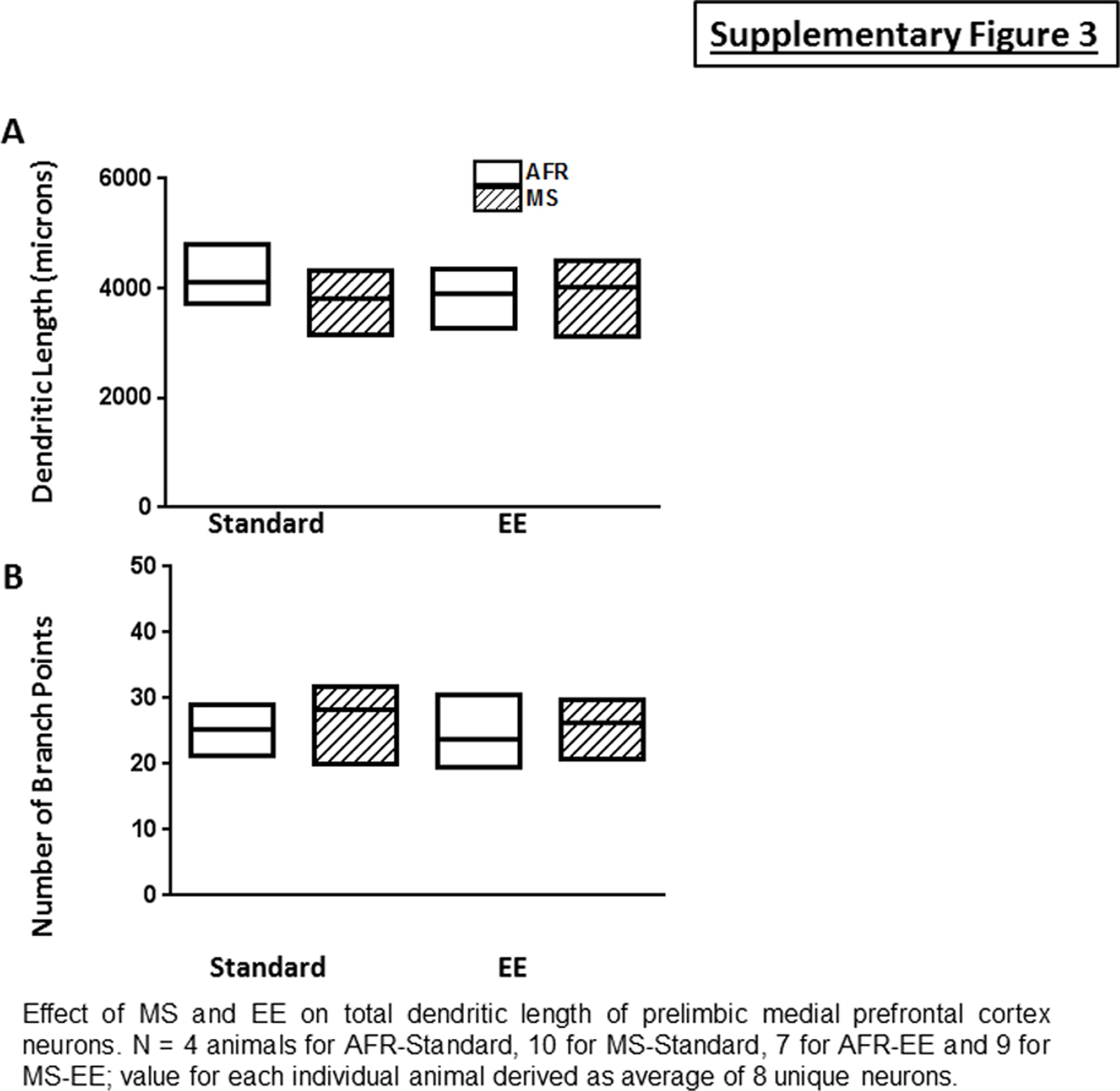

Supplement: Supplementary Figure 3 [file tp2015217x3.tif]

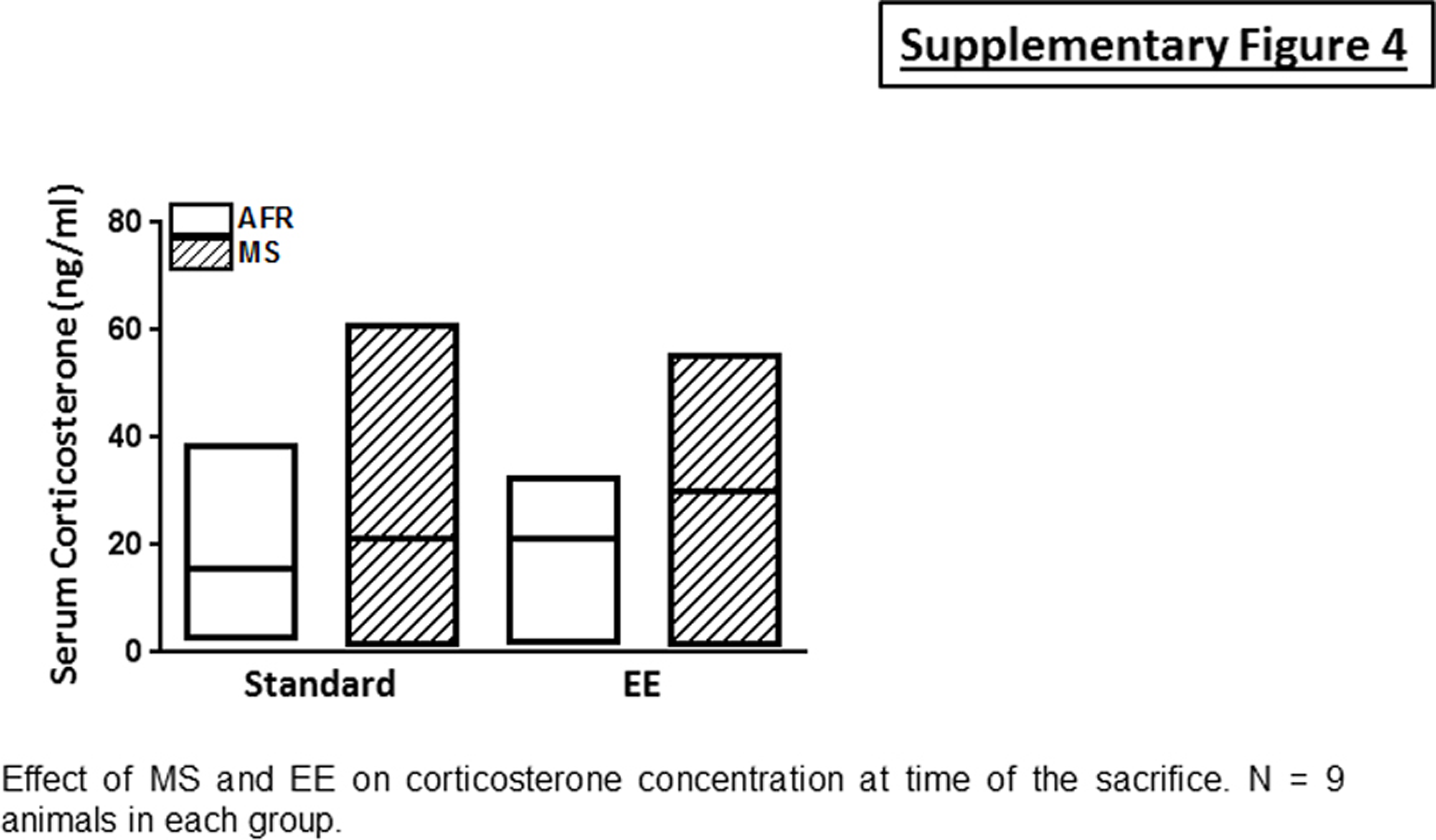

Supplement: Supplementary Figure 4 [file tp2015217x4.tif]
